# Combined impact of biosynthesized selenium nanoparticles and imipenem against carbapenem-resistant *Pseudomonas aeruginosa* and their associated virulence factors

**DOI:** 10.1186/s12866-025-03932-6

**Published:** 2025-04-23

**Authors:** Mohamed Shawky, Mohamed H. Kalaba, Gamal M. El-Sherbiny

**Affiliations:** Botany and Microbiology Department, Faculty of Science (Boys), Al-Azhar University, Cairo, 11884 Egypt

**Keywords:** CRPA, *P. aeruginosa*, Selenium nanoparticles, Antibiotic combination, Time kill, Antibiofilm, *Bap* and *OmpA* genes

## Abstract

**Background:**

Carbapenem-resistant *P. aeruginosa* (CRPA) is a significant nosocomial pathogen characterized by extensive antibiotic resistance, representing a serious public health concern. It is regarded as a high-priority target for antibacterial research. This study aimed to isolate and identify CRPA isolates and the biosynthesis of selenium nanoparticles (Se-NPs) as a novel therapeutic approach for combating CRPA strains and their capacity to form biofilms, alone or in combination with imipenem.

**Methods:**

CRPA isolates were isolated from different clinical samples, identified, and subjected to antibiotic profiling using Vitek-2 method. The detection of biofilm was performed using Congo red agar (CRA), Microdilution broth assay (MBA), and qRT-PCR detection of *Bap* and *ompA* genes. Biosynthesis of Se-NPs with a cell-free filter (CFF) of *Streptomyces* sp. was done and characterized with various techniques, including UV-Vis, XRD, TEM, FTIR, and Zeta potential measurement. The antibacterial efficacy and minimum inhibitory concentrations (MICs) were determined using disc diffusion and microdilution techniques. The checkerboard assay was used to formulate various combinations of imipenem and Se-NPs, alongside time-kill assays to assess their antimicrobial efficacy. Furthermore, the cytotoxic effects and hemolytic activity of Se-NPs, imipenem and their combination were assessed.

**Results:**

The identification process and antibiotic susceptibility testing confirmed that the bacterial isolates were found to be CRPA. Phenotypic analysis revealed that the CRPA produced biofilm, and qRT-PCR demonstrated that all CRPA strains under study have the *Bap* and *ompA* genes. The CFF of *Streptomyces* sp. was able to biosynthesize Se-NPs which presented UV-Visible spectrometric profile with sharp peak at 290 nm. Se-NPs appeared to be a spherical shape, with particle sizes ranging from 20 to 100 nm under TEM and have zeta potential value of -40 mV. The MICs of Se-NPs and imipenem ranged from 6 to 14 and 12 to 14 µg/ml, respectively. The fractional inhibitory concentration index (FICI) values ranged from 0.37 to 0.50 against tested CRPA strain with a significant reduction in the concentrations of Se-NPs and imipenem. QRT-PCR showed that Se-NPs alone or combination of Se-NPs and imipenem led to a reduction of *Bap* and *ompA* gene expression compared to control (*p* ≤ 0.0001). The study showed a significant difference in cell viability was observed across normal or cancer cell lines at high concentrations. However, the combination of Se-NPs and imipenem demonstrated enhanced selectivity toward cancer cells, with HepG-2 cells showing significantly lower viability compared to normal HFP-4 cells across all tested concentrations. Se-NPs alone showed moderate hemolysis percentages of 1.9% at 12 h and 2.3% at 24 h while the hemolytic activity Se-NPs and imipenem combination was reduced to 1.4% and 1.7% at 12 and 24 h, representing approximately 26% and 26% reductions in haemolysis compared to Se-NPs alone at the respective time points.

**Conclusion:**

This study confirms that the biosynthesized Se-NPs exhibit potent synergistic effects with imipenem against CRPA, significantly reducing biofilm formation and the expression of virulence genes *Bap* and *ompA.*

## Introduction

Carbapenem-resistant *P. aeruginosa* is an opportunistic nosocomial pathogen that poses a significant risk, leading to considerable morbidity and mortality. The available treatment options for CRPA are limited and hindered by pharmacokinetic limitations, such as high toxicity and inadequate plasma levels. Therefore, the World Health Organization has designated CRPA as a top-priority pathogen, emphasizing the urgent need for investment in developing new drugs to address this critical healthcare challenge [1–2]. In this context, *Pseudomonas aeruginosa* (*P. aeruginosa*) exhibits a versatile and significant resistance to carbapenems, which have long been recognized as the antibiotics of last resort for managing resistant infections. The organism can develop multiple and persistent resistance mechanisms to carbapenems, including the synthesis of carbapenemases, which are a broad category of β-lactamases capable of hydrolyzing nearly all β-lactam antibiotics, including carbapenems. *P. aeruginosa* can produce carbapenemases from various molecular classes, including chromosomal, plasmid-encoded, and those mediated by integrons or transposons. These enzymes encompass Ambler class A (such as KPC and certain GES variants), class B (including various metallo-β-lactamases like IMP, VIM, and NDM), and class D (oxacillinases with the ability to hydrolyze carbapenems, such as OXA-198). Furthermore, the derepressing of chromosomal AmpC cephalosporinases in *P. aeruginosa* plays a role in enhancing carbapenem resistance, particularly when combined with other mechanisms such as reduced permeability or overexpression of efflux pumps [2].

Furthermore, *Pseudomonas* infections pose distinct challenges in their treatment, particularly when they develop into biofilm structures. These biofilms facilitate the spread of infections among individuals and contribute to outbreaks that are challenging to manage. Biofilm is composed of clusters of microorganisms embedded within an extracellular polymeric substance (EPS) matrix, providing a protective environment that significantly increases the bacteria’s resistance to antibiotics and the innate immune responses of the host. This complicates the effective treatment of *P. aeruginosa* [3]. The roles of outer membrane proteins (OMPs) and the biofilm-associated protein (*Bap*) are critical in the biofilm formation of CRPA. These elements are essential to the bacterium’s pathogenicity and its ability to resist antibiotics. Notably, the overexpression of outer membrane protein A (*ompA*) with his family *(OprF*,* OprL*,* PA0833*, and *PA1048*) has been recognized as an independent risk factor for heightened mortality rates linked to nosocomial pneumonia and bacteremia caused by *P. aeruginosa*. Biofilm development significantly increases the ability of *P. aeruginosa* to adhere to and persist on both biological tissues and non-living surfaces. The formation of pili and the production of the *Bap* surface-adhesion protein are critical components in the biofilm formation process, serving as key contributors to the worsening of *P. aeruginosa* infections [4–6]. The increasing prevalence of antibiotic resistance has led researchers to explore nanotechnology-driven alternatives for antimicrobial therapies. Nanoparticles (NPs) offer unique advantages in drug delivery due to their small size, large surface area-to-volume ratio, and versatile surface chemistry. These exceptional characteristics of NPs improve their interactions with biological entities, including bacteria, when compared to traditional microparticles [7].

Selenium, known as a crucial micronutrient, plays an indispensable role in human health and is often examined for disease management. Furthermore, it exhibits a variety of advantageous properties, such as antioxidative effects, regulation of the immune system, antibacterial and antifungal activities, cancer prevention, enhancement of growth performance, and improvements in reproductive health [8]. While trace amounts of selenium are effective for its anti-cancer and antimicrobial properties, excessive consumption can result in toxicity [9]. Selenium nanoparticles exhibit a diverse array of physical, chemical, electrical, optical, magnetic, semi-conductive, catalytic, and biological attributes [8–11]. Furthermore, Se-NPs provide enhanced bioavailability, lower toxicity, and improved absorption compared to their inorganic and organic forms, rendering them significant in medical diagnostics and nanobiotechnology [12]. Studies suggest that Se-NPs have considerable potential for use in the biomedical sector, encompassing applications in antimicrobial, anti-parasitic, anti-diabetic, drug and gene delivery, as well as anti-cancer therapies. Se-NPs are recognized for their ability to activate glutathione S-transferase (GST) and stimulate the production of reactive oxygen species (ROS), which hinders the maturation of cancer cells [13–15]. The anticancer and antimicrobial activities of metal and metal oxide nanoparticles involve several mechanisms, including direct damage to cellular membranes, the inhibition and disruption of biofilm formation, the production of reactive oxygen species (ROS) and reactive nitrogen species (RNS), and the denaturation of essential biological macromolecules such as nucleic acids and proteins.

The sustainable technique for synthesizing NPs represents an innovative alternative to established chemical and physical methods. This unified research strategy utilizes the benefits of NPs synthesis, which is valued for its straightforwardness, biocompatibility, safety, and affordability. Recent studies have indicated various biological sources that can be employed for this synthesis. Specific organisms, including plants, fungi, actinomycetes, and bacteria, can effectively transform toxic ions into less harmful substances, such as sediments or nanoparticles [8, 16–17].

The utilization of high concentrations of NPs necessary for effective antibacterial performance may lead to reduced biocompatibility and heightened cytotoxicity in physiological environments. To mitigate this concern, enhancing the NPs surface with biocompatible antibacterial agents emerges as a viable approach. The addition of antibiotics can produce a synergistic effect when paired with metal or metal oxide NPs at safe, low concentrations. For instance, a partial synergistic effect was observed when Ag/AgCl NPs were combined with vancomycin, erythromycin, or gentamicin against *P. aeruginosa*, *Klebsiella pneumoniae (K. pneumoniae)*, and *Escherichia coli (E. coli)*. Also, the combination of CuO nanoparticles with various antibiotics demonstrated the most significant synergistic effect reduction of MICs for CuO nanoparticles and antibiotics against methicillin-resistant *Staphylococcus aureus* (MRSA) [18]. In this regard, Zeraatkar et al. [ [19], demonstrated that Se-NPs augment the potency of standard antibiotics against multidrug-resistant *P. aeruginosa* and *Acinetobacter baumannii* (*A. baumannii*) through a synergistic action.

The innovative aspects of this study are the eco-friendly synthesis of Se-NPs using CFF of *Streptomyces* sp. and subsequently employing them to combat CRPA. Furthermore, we compared the effects of these biosynthesized nanoparticles with those of traditional antibacterial antibiotics on the growth of different CRPA strains. Investigated the synergistic efficacy of combination Se-NPs with imipenem against CRPA strains. Evaluate the multifunctional capabilities of Se-NPs, including their ability to inhibit biofilm formation and their role in downregulating genes associated with clinical strains of CRPA as well as hemocompatibility. Therefore, this study aimed to investigate the in vitro antibacterial effects of biosynthesized Se-NPs, both independently and in combination with imipenem, on isolates of carbapenem-resistant *P. aeruginosa*.

## Materials and methods

### Identification and antibiotic profiling of clinical isolates

In this study, nine clinical bacterial isolates, labeled PA-1 to PA-9, were collected from sputum, blood, urine, and abscess pus, at a private laboratory located in Cairo, Egypt, from January to April 2024. The Institutional Review Board of National Cancer Institute (NCI), Cairo University, Egypt, authorized all protocols and procedures (IRB number: IRB00004025; approval number: CB2309-302-071). Each participant signed their written informed consent form before enrolling in this study. The isolates were subject to identification and antibiotic susceptibility testing through the automated Vitek2 system (GN-card) Version 05.04, produced by BioMerieux SA, France [20].

### Determination of biofilm formation by CRPA isolates

#### Qualitative assessment via congo red agar (CRA)

The assessment of biofilm formation by *P. aeruginosa* isolates was conducted using CRA medium (g/l): 37.0 of brain heart infusion (Difco), 50.0 of sucrose, 10.0 of agar, and 0.8 of Congo red (Sigma-Aldrich, Germany). Bacterial isolates were inoculated onto the CRA plates and incubated for 24 h at a temperature of 37 °C. Upon completion of the incubation, the bacterial isolates that produced biofilms exhibited black colonies, while non-biofilm-forming bacteria resulted in red colonies [21].

#### Semi-quantitative assessment via microdilution broth assay (MBA)

The assessment of biofilm formation was conducted semi-quantitatively utilizing 96-well microplates. Each bacterial isolate was grown in Trypticase Soy Broth (TSB) (Oxoid, UK) supplemented with 1% glucose for 24 h at a temperature of 37 °C. Following this incubation, diluted cultures were introduced into the wells of the microplate. Upon completion of the incubation period, the culture media and plates underwent washing with phosphate-buffered saline (PBS) at pH 7.2 to remove any non-adherent bacteria. The biofilms that adhered to the wells were subsequently fixed, dried, and stained using crystal violet (Oxford, UK). The stained biofilms were then solubilized with 95% ethanol, and their optical density (OD) was measured at a wavelength of 492 nm using a plate reader, with each measurement performed in triplicate [22]. The classification of the bacteria that produced biofilms was based on the data presented in Table 1.

**Table 1:** Classification of bacteria based on biofilm formation capacity

Biofilm Class	Results
OD > 4×ODc	Strong biofilm
2×ODc ˂ OD ≤ 4×ODc	Medium biofilm
ODc ˂ OD ≤ 2×ODc	Poor biofilm
OD ≤ ODc	Negative biofilm

#### Detection of biofilm formation genes *Bap* and *OmpA*

The investigation of the expression of *Bap* and *ompA* operon genes, which play a role in biofilm formation in multidrug-resistant bacteria, was conducted through the qRT-PCR technique. For the extraction of RNA and synthesis of complementary DNA (cDNA), (Roche, Basel, Switzerland), was utilized. A Nanodrop spectrophotometer (Thermo Scientific, Waltham, MA, USA) to measure the extracted genome at A260/A280 nm. The ratio obtained was 1.85, which reflects the purity of the DNA. After RNA extraction and cDNA synthesis, the expression of the target genes was analyzed through the qRT-PCR technique. The reaction mixture consisted of 12.5 µL of 2X SYBR green master mix (Applied Biosystems, USA), 1 µL of each forward and reverse primer (as detailed in Table 2), and 5.5 µL of cDNA, culminating in a total volume of 20 µL. The thermal cycling protocol included an initial denaturation at 95 °C for 4 min, followed by denaturation at 94 °C for 10 s, primer annealing at 58 °C for 20 s, and extension at 72 °C for 25 s, with this cycle being repeated 30 times [23–24]. The reference gene for housekeeping was *rpoB*, which was utilized for data analysis, and melting curve analysis was performed on the replicates of each gene to ensure primer specificity. The *Bap* and *ompA* genes relative expression levels were evaluated using the comparative critical threshold 2^−ΔΔCT^ method [25]. The expression level differences were analyzed via two-way ANOVA applied through GraphPad Prism 9.00 software. Statistical significance was established at p-values of ≤ 0.05.

**Table 2:** Oligonucleotides sequences used in this study, *Bap*: biofilm associated protein, *ompA*: outer membrane protein A and housekeeping gene *RpoB*

Genes	Primer sequences 5’-3	(bp) Product size	Ref
*rpoB*	ACGCCTAAAGGTGAAACTCAGTTAA GTACCAGATGGAACACGTAAAGATG	110	[25]
*Bap*	F: GCCAGCGATGTATTGGTAGT R: GGCTCAGCTGTTCCACTAAA	107	[26]
*ompA*	F: TCTTGGTGGTCACTTGAAGC R: ACTCTTGTGGTTGTGGAGCA	86	[27]

*F: forward, R: reverse

#### Green synthesis of Se-NPs by *Streptomyces* Sp.

*Streptomyces* sp. obtained from Bacteriology Lab. Botany and Microbiology Department, Faculty of Science, Al-Azhar University, Egypt, was used to synthesize Se-NPs extracellularly [28]. This strain was inoculated into 250 ml flasks containing 50 ml of ISP2 medium, which comprised the following components (g/l): 4.0 dextrose, 4.0 yeast extract, and 10.0 malt extract, adjusted to a pH of 7.2. The inoculated flasks were incubated for seven days at a temperature range of 30–32 °C while being agitated in a rotary shaker at 200 rpm. Following incubation, the cultures underwent centrifugation at 12,000 rpm for 15 min to separate the mycelium and planktonic cells and to obtain cell-free filtrate (CFF). Subsequently, 50 ml of a 4 mM sodium selenate (Na_2_SeO_4_) aqueous solution (Sigma Aldrich, USA) was combined with CFF (50 ml), and the resulting reaction mixtures were incubated at 37 °C for 48 h in a dark condition. The production of Se-NPs was visually indicated by a colour transition of the solution from pale yellow to red [29, 30]. As a result, the deep orange colloidal supernatant which represent the reaction mixture of the promising strain and sodium selenatewas selected for further analysis. A portion of this solution was centrifuged for 30 min at 8000 rpm to separate the Se-NPs, which were then dried at 70 °C until constant weight was obtained for further studies.

#### Characterization of biosynthesized nanoparticles

The biosynthesis of selenium nanoparticles was validated by the presence of a distinct peak observed in the absorption spectrum of the solution, as determined through UV-visible spectroscopy utilizing a JASCO V630 spectrophotometer [31]. The dried Se-NPs were analyzed for their X-ray diffraction spectra using the XRD-6000 Shimadzu device from Japan. A conventional Theta/2Theta diffractometer, which utilizes a copper X-ray source, was employed for the analysis. The scans were conducted at a speed of 2 degrees per minute, with the diffraction angle ranging from 10 to 80 degrees. The apparent crystal size (ACS) of the Se-NPs was calculated according to the Debye-Scherrer equation, which is formulated as follows:

$$ACS = Kλ/({\beta} cos(θ))$$

Where K is the shape factor, generally taken as 0.9, λ represents the wavelength of the X-ray, β is the full width at half maximum measured in radians, and θ is the diffraction angle [32]. For the functional characterization of Se-NPs, the Fourier transform infrared spectroscopy (FTIR) was performed using the FTIR 6100 spectrometer (Jasco, Japan). The nanoparticles were ground with potassium bromide (KBr) before analysis. Subsequently, the infrared spectra were recorded in the wavenumber range of 4000–400 cm^− 1^ using a Bruker Vertex 80 V with a resolution of approximately 4–8 cm^− 1^ [33]. The structural characteristics were examined through transmission electron microscopy (TEM) imaging at 80 kV at the Regional Center for Mycology and Biotechnology at Al-Azhar University. For sample preparation, a drop of the solution was placed on carbon-coated copper grids (CCG) and allowed to drain slowly at room temperature before capturing the TEM micrograph [34]. The zeta potential of selenium nanoparticles was detected using a Zetasizer Nano Particle Analyzer (Malvern Instruments Ltd., UK) [35].

### Antibacterial activity of biosynthesized Se-NPs

The antibacterial efficacy of biosynthesized Se-NPs against CRPA strains was evaluated using the disc diffusion method following Clinical and Laboratory Standards Institute (CLSI) guidelines (CLSI) [20].

### Minimum inhibitory concentration of biosynthesized Se-NPs

The minimum inhibitory concentration of biosynthesized Se-NPs against CRPA isolates was determined utilizing the microbroth dilution method, following CLSI, 2023 guidelines [20]. berifly, bacterial strains were cultured overnight to reach the stationary phase at 37 °C with aeration at 180 rpm, followed by dilution in Mueller–Hinton broth (MHB) to achieve a cell density of approximately 1.0 × 10^− 6^ colony-forming units (CFU/mL). Subsequently, 100 µl aliquots of this suspension were transferred into a UV-sterilized polystyrene 96-well microtiter plate (SPL, Korea), which contained 100 µl of the serially diluted fabricated Se-NPs solution (0–100 µg/ml), imipenem was used as a positive control. (0–20 µg/ml) and well contains MHB as a negative control. After incubating the plates at 37 °C for 24 h, the MIC was identified as the lowest concentration at which no visible bacterial growth was observed.

### Checkerboard assay

A checkerboard assay was conducted on a 96-well plate to evaluate the combined effects of Se-NPs and imipenem, utilizing a bacterial isolate (PA-9) of CRPA as the experimental model. This study systematically varied the concentrations of Se-NPs in a two-fold dilution across the columns. In contrast, the imipenem concentrations were modified along the rows starting the dilution process with the MIC value of it. After a 24-hour incubation period at 37 °C, the MIC was determined using a microplate reader (BioTek Instruments, Winooski, USA) at a wavelength of 620 nm. The FICI, which assesses the interactions between the two agents as synergistic, additive, or antagonistic, was calculated using the formula: FICI = (MIC of combination / MIC Se-NPs) + (MIC of combination / MIC imipenem). A FICI value of 0.50 or lower indicates synergy, while values ranging from 0.50 to 1.00 suggest an additive effect. Values between 1.00 and 2.00 reflect indifference, and any FICI exceeding 2.0 indicates antagonism.

### Time-kill curves evaluation

Time kill assay was performed to assess the effect of Se-NPs-imipenem synergistic combination at different time intervals. Briefly, overnight cultures of the bacteria were cultivated in nutrient broth, which was derived from a single colony on brain heart infusion agar. The CRPA (PA-9) isolate (0.5 McFarland) was treated at concentrations of ¼ MIC, ½ MIC, ¾ MIC, 1 MIC, and 2 MIC of the Se-NPs- imipenem synergistic combination, as established through the checkerboard assay. At designated time points over 24 h, 100 µl aliquots were collected, plated onto nutrient agar, and incubated for 18–24 h. After incubation, the colony-forming units (CFUs) were counted, enabling the construction of a time-kill curve to assess the antimicrobial activity (CFU/ml decline concerning the initial inoculum) [36].

### Antibiofilm properties of Se-NPs and Imipenem -Se-NPs

Following the determination of the MICs of Se-NPs, a value of ¾ MIC (sub-MIC) was employed to evaluate the antibiofilm effectiveness against CRPA isolates using the methodology previously outlined for crystal violet. In addition, the ¾ MIC (sub-MIC) of combination imipenem -Se-NPs, was used for the detection of biofilm gene expression using the bacterial isolate (PA-9) as a model via the method previously mentioned in the biofilm formation section. Control wells included those with Se-NPs-free culture medium, combination imipenem -Se-NPs at the same tested concentrations and those containing culture medium inoculated solely with the tested bacterial strains.

### In vitro cytotoxicity assessment

To evaluate the cytotoxic effects of the biosynthesized Se-NPs and their combination with imipenem in vitro, HFB-4 (normal human melanocytes) cell lines and HepG-2 (hepatocellular carcinoma), were cultured in medium enriched with 10% fetal bovine serum and 1% penicillin-streptomycin (10000 U/ml) at 37 °C in a 5% CO_2_ atmosphere. Once the cells reached 80% confluence, Se-NPs and Se-NPs combined with imipenem were added to the cultures at different concentrations and maintained under humidified conditions for 24 h at 37 °C with 5% CO_2_. The MTT assay (3-(4,5-dimethylthiazol-2-yl)-2,5-diphenyltetrazolium bromide) from (Sigma-Aldrich), was employed to ascertain the 50% inhibitory concentration at wavelengths of 570–630 nm.

### Evaluation of haemolytic activity

The haemolytic potential of the formulated Se-NPs and their combination with imipenem were assessed for cytocompatibility using healthy fresh human red blood cells (RBCs). In this experiment, RBCs were exposed to the prepared Se-NPs and their combination with imipenem at a ratio of 10:1 in MIC. For control purposes, 1% Triton X-100 was utilized as the positive control, while phosphate-buffered saline (PBS) served as the negative control. Following an incubation period of 12 and 24 h at 37 °C, the cells were subjected to centrifugation at 3000×g for 10 min, and the absorbance of haemoglobin released into the supernatants was measured at 570 nm using a spectrophotometer (BioTek Instruments Winooski, USA). The relative haemolysis was calculated using the formula:

Homolysis (%) = [A Sample– A Blank / A Positive Control– A Blank] x100 [37].

### Statistical analysis

Experiments were carried out in triplicate, and the results are presented as mean ± standard deviation. A two-way ANOVA was employed to compare experimental and control groups. Statistical computations were conducted using GraphPad Prism Software version 8.0 (GraphPad Software, Inc, La Jolla, CA, USA) to determine statistically significant differences (p-value < 0.05).

## Results and discussion

### Isolation, identification, and antibiotic profile of *P. aeruginosa* isolates

The challenge of drug resistance presents a significant barrier in the treatment of microbial infections, leading researchers to explore novel strategies to combat resistant microorganisms. Nanotechnology has been identified for its ability to combat bacterial infections and contribute to the reduction of drug resistance. In addition to their antibacterial properties, these agents offer further benefits to the body by promoting microbial balance and improving immune response [23]. In this study, the nine bacterial isolates were identified as *P. aeruginosa* with a high probability of 96–98% using the Vitek-2 automated system, as detailed in Table 3. The susceptibility of these bacterial isolates was assessed against fifteen antibiotics from various classes. The findings revealed a significant prevalence of extensive drug resistance among the tested *P. aeruginosa* isolates. This study classified the nine *P. aeruginosa* isolates as CRPA; however, these isolates exhibited sensitivity to amikacin, and aztreonam, as shown in Table 4. This finding is consistent with previous studies, confirming that all *P. aeruginosa* isolates, accounting for 100%, exhibited resistance to eight specific antibiotics: ceftazidime, levofloxacin, amikacin, gentamicin, netilmicin, piperacillin, ciprofloxacin, and tobramycin. Additionally, it was observed that 87% of the *P. aeruginosa* isolates exhibited resistance to both ofloxacin and aztreonam [38]. The outer membrane of *P. aeruginosa* serves as a distinct barrier that restricts the penetration of antibiotics. This membrane is primarily made up of a bilayer of phospholipid molecules, lipopolysaccharides (LPS), and porins that are integrated within the phospholipids. It plays a crucial role in the selective and non-selective uptake of external substances, which is facilitated by various porin types. These include non-specific porins such as OprF, specific porins like OprB, OprD, OprE, OprO, and OprP, gated porins such as OprC and OprH, as well as efflux porins like OprM, OprN, and OprJ [39]. Furthermore, *P. aeruginosa* is notably classified as one of the multidrug-resistant (MDR) ESKAPE pathogens, which include *Enterococcus faecium*,* S. aureus*,* K. pneumoniae*,* A. baumannii*,* P. aeruginosa*, and *Enterobacte*r. In this regard, the World Health Organization (WHO) has categorized carbapenem-resistant *P. aeruginosa* within the “critical” group of pathogens, indicating an urgent need for the development of new antibiotics for clinical use [40–41].

**Table 3:** Identification of the carbapenem-resistant bacterial isolates by Vitek2 system

Isolate code	Name of Bacteria	Confidence level	% Probability
PA-1	*P. aeruginosa*	Excellent	98%
PA-2	*P. aeruginosa*	Excellent	98%
PA-3	*P. aeruginosa*	Excellent	98%
PA-4	*P. aeruginosa*	Excellent	98%
PA-5	*P. aeruginosa*	Excellent	96%
PA-6	*P. aeruginosa*	Excellent	96%
PA-7	*P. aeruginosa*	Excellent	98%
PA-8	*P. aeruginosa*	Excellent	98%
PA-9	*P. aeruginosa*	Excellent	98%

**Table 4:** Antibiotic susceptibility testing of CRPA isolates

Antibiotics	PA-1		PA-2		PA-3		PA-4		PA-5		PA-6		PA-7		PA-8		PA-9	
	MIC	Profile	MIC	Profile	MIC	Profile	MIC	Profile	MIC	Profile	MIC	Profile	MIC	Profile	MIC	Profile	MIC	Profile
Ciprofloxacin	≥ 2	R	≥ 2	R	≥ 2	R	≥ 2	R	≥ 2	R	≥ 2	R	≥ 2	R	≥ 2	R	≥ 2	R
Levofloxacin	≥ 4	R	≥ 4	R	≥ 4	R	≥ 4	R	≥ 4	R	≥ 4	R	≥ 4	R	≥ 4	R	≥ 4	R
Aztreonam	< 32	S	< 32	S	< 32	S	< 32	S	< 32	S	< 32	S	< 32	S	< 32	S	< 32	S
Gentamicin	≥ 16	R	≥ 16	R	≥ 16	R	≥ 16	R	≥ 16	R	≥ 16	R	≥ 16	R	≥ 16	R	≥ 16	R
Ampicillin	≥ 16	R	≥ 16	R	≥ 16	R	≥ 16	R	≥ 16	R	≥ 16	R	≥ 16	R	≥ 16	R	≥ 16	R
Ampicillin/Sulbactam	≥ 32	R	≥ 32	R	≥ 32	R	≥ 32	R	≥ 32	R	≥ 32	R	≥ 32	R	≥ 32	R	≥ 32	R
Amikacin	< 64	S	< 64	S	< 64	S	< 64	S	< 64	S	< 64	S	< 64	S	< 64	S	< 64	S
Ceftazidime	> 32	R	> 32	R	> 32	R	> 32	R	> 32	R	> 32	R	> 32	R	> 32	R	> 32	R
Cefepime	> 32	R	> 32	R	> 32	R	> 32	R	> 32	R	> 32	R	> 32	R	> 32	R	> 32	R
Tobramycin	≥ 16	R	≥ 16	R	≥ 16	R	≥ 16	R	≥ 16	R	≥ 16	R	≥ 16	R	≥ 16	R	≥ 16	R
Piperacillin/Tazobactam	> 4	R	> 4	R	> 4	R	> 4	R	> 4	R	> 4	R	> 4	R	> 4	R	> 4	R
Imipenem	> 8	R	> 8	R	> 8	R	> 8	R	> 8	R	> 8	R	> 8	R	> 8	R	> 8	R
Meropenem	> 8	R	> 8	R	> 8	R	> 8	R	> 8	R	> 8	R	> 8	R	> 8	R	> 8	R
Polymyxin B	> 2	R	< 2	S	> 2	R	> 2	R	> 2	R	> 2	R	< 2	S	> 2	R	< 2	S
Colistin	> 2	R	< 2	S	> 2	R	> 2	R	> 2	R	> 2	R	< 2	S	> 2	R	< 2	S

### Characterization of biosynthesized Se-NPs

**Fig. 1:**
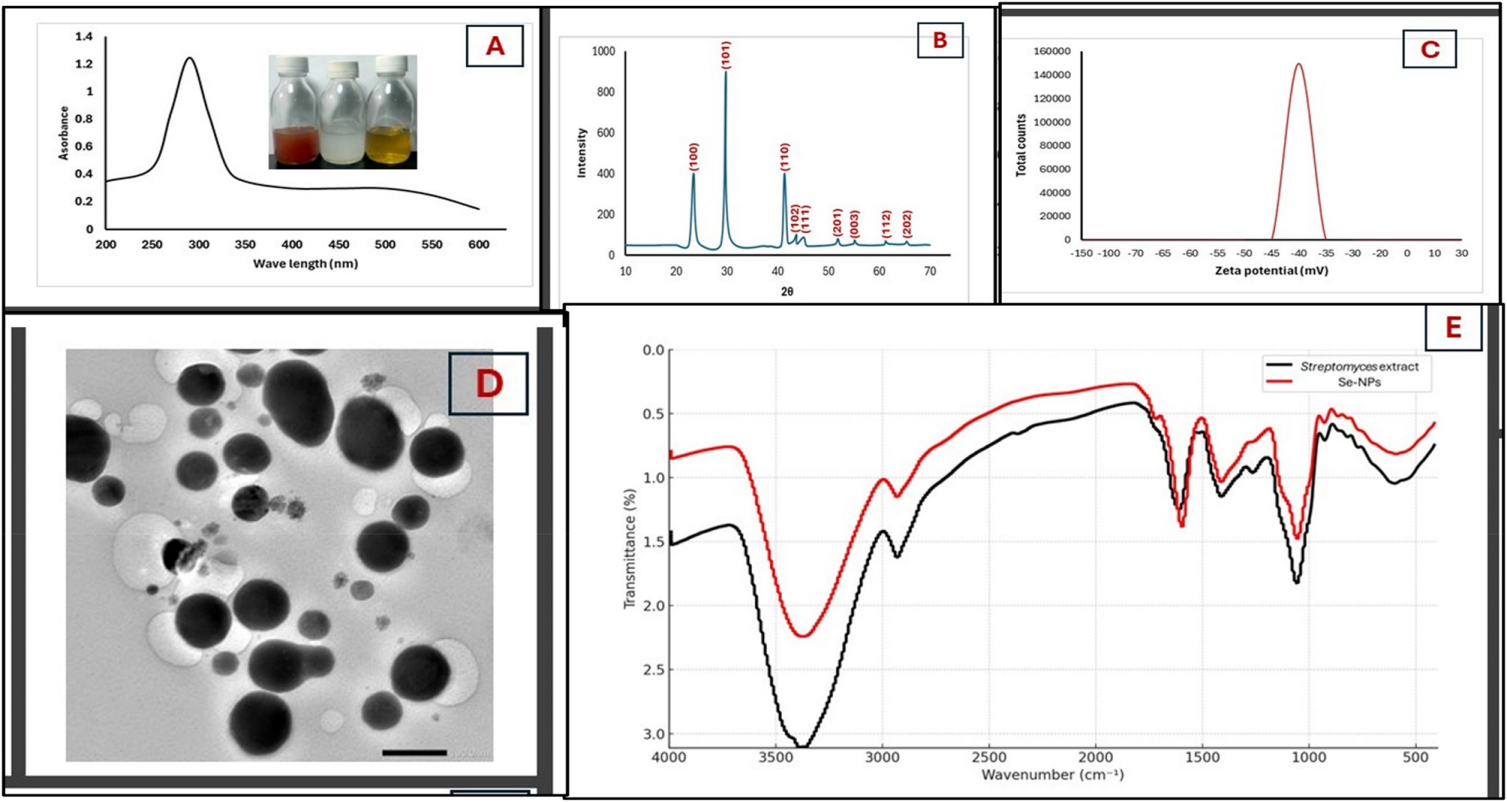
Biosynthesis and characterization of Se-NPs, (A) UV-visible, (B) X-ray diffraction pattern (XRD), (C) Zeta potential, (D) transmission electron microscopic (TEM), and (E) FTIR spectrum.

*Streptomyces* species demonstrated their capability to function as biological factories for the successful synthesis of nanoparticles. Hassan et al. [42], utilized a cell-free filtrate derived from *Streptomyces parvulus* MAR4 for the biosynthesis of selenium nanoparticles. Similarly, Shaaban et al. [43], employed a cell-free filtrate from *Streptomyces enissocaesilis* for the same purpose of synthesizing selenium nanoparticles. In this study, the biosynthesis of Se-NPs was conducted using a cell-free filtrate obtained from *Streptomyces* species. The initial indication of Se-NPs synthesis was observed through a color change in the medium, which shifted to red as sodium selenate (Na_2_SeO_4_) was reduced to elemental selenium (Se^0^). The reduction process was confirmed by UV–visible spectroscopy, which validated the conversion of selenite (Na_2_SeO_4_) to Se^0^ facilitated by the cell-free filtrate from *Streptomyces* sp. Selenium nanoparticles (Se-NPs) formation was confirmed by the prominent absorption peak observed at 290 nm in the UV-visible spectrum, which is characteristic of selenium nanostructures, as shown in Fig. 1A. These findings are consistent with an earlier investigation carried out by Thamayandhi and his colleagues, which emphasized UV absorption of Se-NPs at 270–300 nm [40]. The pronounced peak observed at 290 nm suggests the presence of selenium nanoparticles, and this finding closely resembles the results reported by Vahdati and Tohidi Moghadam [44] as well as those by Yin and Meng [45]. X-ray diffraction (XRD) analysis (Fig. 1B) provided definitive evidence of the crystalline nature of the biosynthesized Se-NPs, revealing distinct diffraction patterns with peaks at 2θ values of 23.5, 29.7, 41.3, 43.6,,45.5, 51.8, 55.2, 61.2, and 65.3°. These peaks correspond to the (100), (101), (110), (102), (111), (201), (003), (112), and (202) crystallographic planes, respectively, which align with the standard reference pattern for hexagonal selenium (JCPDS card No. 06-0362). This confirms that the biosynthesized nanoparticles possess a hexagonal crystalline structure. In this context, Thamayandh et al. [40], reported that the XRD analysis of Se-NPs peaks centered at 2θ values of 24.5◦, 30.0◦, 42.3◦, 45.1◦, 48.7◦, 53.5◦, 60.1◦, and 68.5◦ was related to the crystal planes of (100), (101), (110), (102), (111), (201), (202) and (210). Furthermore, Tugarova and Mamchenkova [46] and Hosseini et al. [47] synthesized Se-NPs from *Streptomyces* sp. and observed diffraction peaks at 2θ (degrees) of 23°, 29°, 41°, 43°, 45°, 51°, 56°, 61°, 65°, and 71°, with an approximate size of 37 nm, which were consistent with the results of this study. The zeta potential measurement of -40 mV indicates excellent colloidal stability of the Se-NPs suspension, as values exceeding ± 30 mV typically denote high electrostatic repulsion between particles, preventing aggregation as illustrated in Fig. 1C. The zeta potential is an important measure that reflects the electrical characteristics of nanoparticles, particularly the charge on their surfaces. Our research findings are consistent with those of Mollania et al. [48], who reported a significant negative charge on the Se-NPs, quantified at -46.86 mV, which suggests that these selenium nanoparticles possess considerable electrical stability. Vekariya et al. [49], recorded a zeta potential of -28.8 mV, while Zare et al. [50], found a zeta potential of -22.9 mV for Se-NPs synthesized using fungi. The negative zeta potential observed in our study agrees with previously reported values for selenium nanoparticles, thereby validating the reliability of our experimental results. Previous studies have consistently shown that selenium nanoparticles exhibit negative zeta potential values, further supporting the accuracy of our measurements. In our study, TEM imaging revealed spherical nanoparticles with size distribution ranging from approximately 20 nm to 100 nm, dispersed without significant aggregation as shown in Fig. 1D. The spherical morphology observed in TEM aligns with the crystalline structure indicated by XRD analysis, providing complementary evidence for the successful synthesis of well-defined Se-NPs. The average size of Se-NPs, as determined by TEM imaging, is greater than the size measured by XRD. This discrepancy may be explained by the presence of substances on the surfaces of the nanoparticles, including capping and stabilizing agents, which can affect the results obtained from TEM imaging. A previous study conducted by Blinov et al. [51], indicated that the analysis of TEM images of Se-NPs, regardless of their charge, exhibited a spherical morphology with average dimensions of approximately 20–30 nm and 40–50 nm for positive and negative charges, respectively. Furthermore, research by Khudier et al. [52], confirmed that the green-synthesized selenium nanoparticles displayed a distinctly spherical shape and maintained a consistent size of 50 ± 1.23 nm. In a similar vein, Shaaban et al. [43], biosynthesized Se-NPs from the cell-free filtrate of *Streptomyces enissocaesilis*, resulting in particle sizes ranging from 20 to 211 nm. The FTIR analysis was utilized to investigate the interaction between Se-NPs and the CFF of *Streptomyces* sp. FTIR analysis was conducted over a wavenumber range of 4000–400 cm⁻¹ for the CFF of *Streptomyces* sp., which were subsequently compared to the biosynthesized Se-NPs as illustrated in Fig. 1E. The FTIR spectra of CFF of *Streptomyces* and the synthesized selenium nanoparticles (Se-NPs) reveal critical insights into the biomolecular interactions during nanoparticle formation. Both spectra exhibit a broad peak near 3400 cm⁻¹, corresponding to O-H and N-H stretching vibrations from hydroxyl and amine groups. The reduced intensity of this peak in the Se-NPs spectrum suggests these functional groups actively participate in selenium reduction and nanoparticle stabilization. The C-H stretching region around 2900 cm⁻¹ shows decreased intensity in the Se-NPs spectrum, may be indicate to aliphatic compounds contribute to the capping mechanism. Meanwhile, the sharp peak at approximately 1650 cm⁻¹, attributed to carbonyl (C = O) stretching in proteins, appears in both spectra with slight modifications, confirming proteins play a significant role in the bioreduction process. The region between 1500–1400 cm⁻¹, representative of C-N stretching and aromatic vibrations, shows variations that imply nitrogen-containing compounds participate in nanoparticle formation. The fingerprint region (1000–500 cm⁻¹) displays notable similarities between the two spectra, proving that biomolecules from the CFF of *Streptomyces* retain their structural integrity while coating the selenium nanoparticles. However, slight peak shifts and intensity changes in this region may indicate the formation of new chemical bonds, potentially including Se-O interactions. These spectral characteristics collectively demonstrate that biomolecules from the CFF of *Streptomyces* function as both reducing and stabilizing agents during selenium nanoparticle synthesis, providing a protective coating that contributes to the excellent colloidal stability observed in zeta potential measurements [8]. The presence of these biomolecules on the nanoparticle surface may also contribute to their biocompatibility and enhanced biological activity. Tugarova and Mamchenkova [46], reported the analysis of the FTIR spectrum for selenium nanoparticles indicated a range from 400 to 4000 cm⁻¹. Notable bands were detected at 3441, 2920, 2858, 1625, 1537, 1324, 1025, and 1032 cm⁻¹. In addition, Bafghi et al. [8], reported the FTIR peaks for biosynthesized Se-NPs using supernatants from *A. flavus*, which appeared at 1406.91, 1624.40, 2714.14, and 3418.15 cm^− 1^. Conversely, the peaks for *C. albicans*-Se-NPs were found at 1412.81, 1631.83, 2949.83, and 3416.38 cm^− 1^.

### Antibacterial efficacy of Se-NPs

Due to the rising versatility of bacterial resistance to antibiotics, there is an increasing focus on the use of metal-based materials as promising substitutes to combat the issue of antibacterial resistance [17]. Selenium ions (Se^+^) and Se-NPs have been employed as antimicrobial agents since ancient times and continue to find extensive application in the food industry and healthcare settings [15]. Consequently, the antibacterial efficacy of Se-NPs against CRPA strains PA-1, PA-2, PA-3, PA-4, PA-5, PA-6, PA-7, PA-8, and PA-9 were assessed using the agar disc diffusion method. The results revealed that Se-NPs exhibit considerable antibacterial efficacy against CRPA isolates, with inhibition zones ranging from 21 ± 03 to 26 ± 08 mm. On the contrary, these CRPA isolates showed complete resistance to imipenem (0.0 mm inhibition), drawing attention to the potential of Se-NPs as an alternative antimicrobial strategy against this pathogen.

**Fig. 2:**
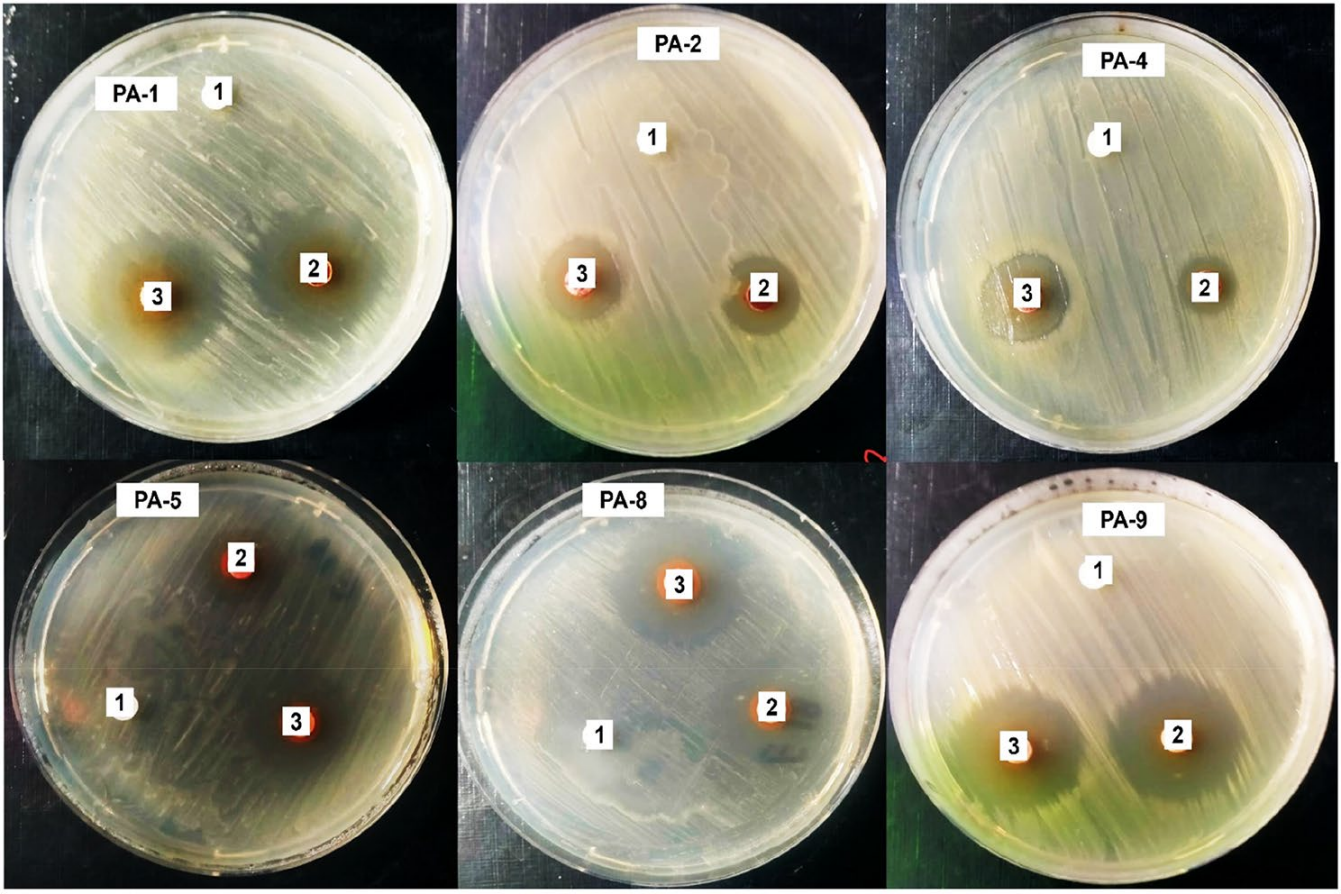
Antibacterial activity (1) imipenem, (2) Se-NPs, and (3) combination imipenem and Se-NPs against some bacterial isolates

The MICs of Se-NPs were found to be between 6 and 14 µg/ml, in contrast to the MICs for imipenem, which ranged from 12.0 to 14.0 µg/ml, as presented in Tables 5 and 6; Fig. 2. The findings of the present study indicated a high effect of biosynthesized Se-NPs against CRPA strains, which was observed to be concentration-dependent. This concentration-dependent efficacy suggests that Se-NPs may operate through mechanisms distinct from conventional antibiotics, potentially involving reactive oxygen species generation, membrane disruption, biofilm inhibition, or enzyme interference. This allows them to overcome resistance mechanisms that render carbapenems ineffective. The primary mechanism is the generation of ROS, which comprises several forms such as peroxides (*O_2_ ^− 2^), superoxide (*O_2_^−^), hydroperoxyl (HO_2_^*^), hydroxyl radical (HO^*^), and singlet oxygen (^1^O_2_^*^), along with RNS like peroxynitrite (ONOO^−^) and nitric oxide (NO^*^). These species are produced under oxidative stress due to the release of metal ions from NPs. Additionally, methodologies such as photodynamic therapy (PDT), chemodynamical therapy (CDT), and sonodynamic therapy (SDT) can enhance the accumulation of ROS within cells through the action of ROS-generating nanoparticles [14–16]. The results of the current study are consistent with an earlier study carried out by Khudier and his colleagues, which highlighted the significant activity of Se-NPs against both standard and clinical strains of Gram-positive bacteria, comparable to certain antibiotics [52–54]. Bio-Se-NPs can also reduce heavy metal accumulation and inhibit the growth of *Aeromonas hydrophilia*, *S aureus*,* Bacillus cereus*, *Listeria monocytogenes*, *E. coli*, and *K. pneumoniae* at low concentrations [55]. Also, Cremonini et al. [56], demonstrated the antibacterial activity of Se-NPs against various bacterial strains, including *P. aeruginosa* PAO1, *Stenotrophomonas maltophilia*,* Achromobacter xylosoxidans* strain *C*,* Burkholderia cenocepacia* strain LMG 16,656, *S. aureus* Mu50 strain, *S. aureus* UR1, *S. epidermidis* ET024, and *S. hemolyticus* UST1 with MIC ranging from 4 to 128 µg/ml.

**Table 5:** Antibacterial activity of biosynthesized Se-NPs against CRPA

Bacterial strains	Mean of inhibition zone diameter (mm) ± SD	
	Se-NPs	Imipenem
PA-1	24 ± 07	0.0
PA-2	21 ± 03	0.0
PA-3	25 ± 0.1	0.0
PA-4	23 ± 05	0.0
PA-5	23 ± 07	0.0
PA-6	23 ± 02	0.0
PA-7	25 ± 0.1	0.0
PA-8	26 ± 08	0.0
PA-9	24 ± 0.1	0.0

**Table 6:** MICs of biosynthesized selenium nanoparticles and imipenem

Bacterial strains	MICs (µg/ml)	
	Se-NPs	Imipenem
PA-1	6.0	14.0
PA-2	7.0	13.0
PA-3	7.0	12.0
PA-4	9.0	12.0
PA-5	10.0	12.0
PA-6	7.0	13.0
PA-7	8.0	14.0
PA-8	14.0	12.0
PA-9	7.0	12.0

### Checkerboard assay

Antimicrobial resistance is increasingly recognized as a significant healthcare challenge, prompting the need for the creation of innovative drug classes through unconventional methods. *P. aeruginosa*, a prevalent pathogen associated with hospital-acquired infections, poses substantial treatment difficulties, even when utilizing the most advanced frontline medication, carbapenems. This pathogen’s capacity to develop resistance to newly introduced small-molecule antibiotics necessitates the exploration of a novel biological strategy, which involves the use of nanoparticle combinations with carbapenems and β-lactams as supplementary treatment to provide a lasting resolution to this issue [4]. The checkerboard method has been employed to create diverse combinations of imipenem and selenium nanoparticles, aiming to enhance their efficacy while reducing the required dosage. This traditional checkerboard approach is a recognized standard for evaluating drug synergy, involving the assessment of two drugs at varying concentrations and combinations to identify the concentration of each agent that results in the minimal synergistic effect on the other. The fractional inhibitory concentrations index (FICi) offers a quantitative framework to measure this interaction. The FICi reflects the degree of interaction between imipenem and Se-NPs in combating CRPA [7]. In this study, the checkerboard assay evaluating combinations of imipenem and Se-NPs against carbapenem-resistant *P. aeruginosa* (CRPA) strain PA-9 revealed varying degrees of interactions among the 28 different concentration combinations tested. Among these combinations, three demonstrated significant synergistic effects with fractional inhibitory concentration indices (FICI) of ≤ 0.5. The most effective synergistic combinations were: 1/4 MIC imipenem + 1/4 MIC Se-NPs (3.0 + 3.5 µg/ml) yielding FICI of 0.5; 1/4 MIC imipenem + 1/8 MIC Se-NPs (3.0 + 1.75 µg/ml) with FICI of 0.375; and 1/8 MIC imipenem + 1/4 MIC Se-NPs (1.5 + 3.5 µg/ml) also with a FICI of 0.375 as shown in Table 7; Fig. 2. The remaining combinations resulted in either additive effects (ten combinations) or indifferent interactions (fifteen combinations). The synergistic combinations demonstrated a remarkable ability to reduce the required therapeutic concentrations of both antimicrobial agents. When used individually, the MICs for imipenem and Se-NPs against the PA-9 strain were 12 µg/ml and 14 µg/ml, respectively. However, in the most effective synergistic combinations, these concentrations were dramatically reduced to as low as 1.5 µg/ml for imipenem and 1.75 µg/ml for Se-NPs. This substantial decrease in the required concentrations represents an 87.5% reduction for both imipenem and Se-NPs. As mentioned previously, selenium nanoparticles generate reactive oxygen species leading to oxidative stress and membrane damage in multidrug-resistant bacteria. Additionally, Se-NPs can disrupt biofilm formation and quorum sensing in *P. aeruginosa*, potentially increasing bacterial susceptibility to antibiotics. The synergistic effect may arise from Se-NPs increasing membrane permeability, thereby facilitating enhanced imipenem penetration, which can compromise bacterial membrane integrity, increasing antibiotic uptake. Alternatively, simultaneous targeting of different bacterial cellular components creates a combined stress that overwhelms bacterial defense mechanisms. This multi-target approach appears particularly effective against CRPA, which typically possesses several resistance mechanisms against β-lactam antibiotics including altered membrane permeability, efflux pumps, and carbapenemase production. Han et al. [57], illustrated that Se-NPs exhibit a synergistic effect when used in combination with linezolid against both methicillin-sensitive *Staphylococcus aureus* (MSSA) and methicillin-resistant *Staphylococcus aureus* (MRSA). Additionally, Golmohammadi et al. [58], demonstrated that the combination of Se-NPs with mupirocin significantly enhances antibacterial activity against MRSA, achieving a threefold reduction in the MIC. Moreover, this combination markedly promotes wound contraction, angiogenesis, fibroblast proliferation, collagen synthesis, hair follicle development, and epidermal growth when compared to the control group (*P* ≤ 0.05). In this regard, the integration of Se-NPs with various antibiotics has shown increased effectiveness against multidrug-resistant bacteria, including *P. aeruginosa*, *S. aureus*, *Enterococcus faecalis*, and *E. coli*. Furthermore, the combination of Se-NPs with levofloxacin results in a 50% reduction in MICs compared to the individual effects of Se-NPs and levofloxacin [38]. The results concerning the MICs and minimal bactericidal concentration (MBC) for eight isolates of *P. aeruginosa* revealed that the combination of Se-NPs with antibiotics such as amikacin, levofloxacin, and piperacillin led to a substantial decrease in the concentration of the antibiotic when combined with Se-NPs, specifically observed to be approximately 10 to 20 times lower than that of the unmodified antibiotics [38]. Additionally, Haji and colleagues reported that the FICI values for the synergy between silver nanoparticles (Ag-NPs) and imipenem against *P. aeruginosa* ranged from 0.13 to 0.5 [59].

**Table 7:** Checkerboard assay of Imipenem and Se-NPs combinations against *P. aeruginosa* PA-9

No	MIC _Imipenem_ +MIC _Se−NPs_	Imipenem +Se-NPs (µg/ml)	FIC _Imipenem_ +FIC _Se−NPs_	FICI	Interpretation
1	1/4MIC + 1/4MIC	3.0 + 3.5	0.25 + 0.25	0.5	synergy
2	1/4MIC + 1/8MIC	3.0 + 1.75	0.25 + 0.125	0.375	synergy
3	1/8MIC + 1/4MIC	1.5 + 3.5	0.125 + 0.25	0.375	synergy

### Time-kill assay

**Fig. 3:**
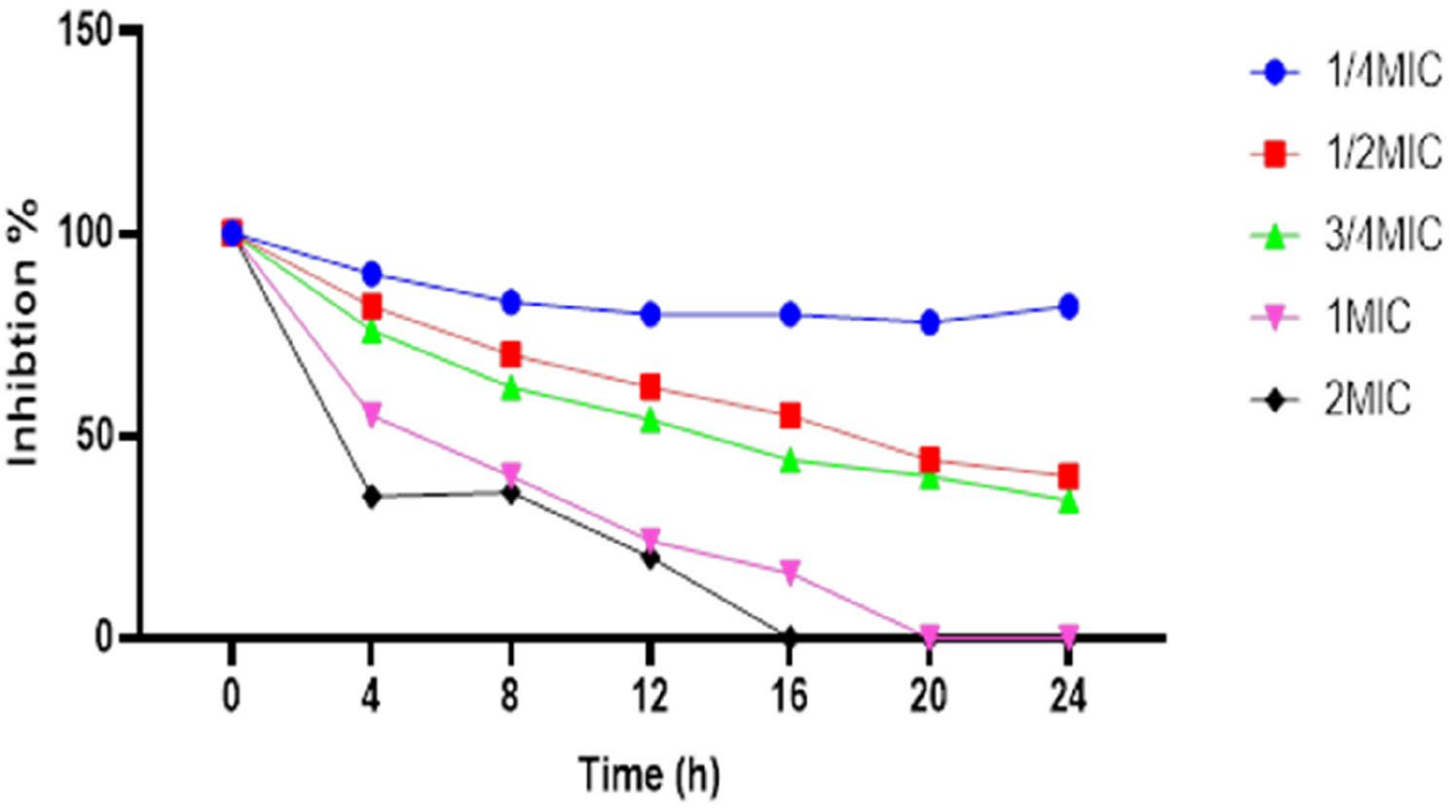
Time-kill curve for Se-NPs at ¼ MIC, ½ MIC, ¾ MIC,1MIC and 2 MIC concentrations, **p < 0.0028, *** *p* < 0.0001

The killing kinetic technique was utilized to determine the minimum time required for the combinations of imipenem and Se-NPs to exert either a bactericidal or bacteriostatic effect on the viability of bacterial cells following treatment. The time-kill assay evaluated the efficacy of the synergistic combination of imipenem and Se-NPs against carbapenem-resistant *P. aeruginosa* (CRPA) strain PA-9 at various concentration levels over 24 h. Five different concentration combinations were tested, representing ¼ MIC (0.35 and 0.87 µg/ml), ½ MIC (0.75 and 1.75 µg/ml), ¾ MIC (1.125 and 2.625 µg/ml), 1 MIC (1.5 and 3.5 µg/ml), and 2 MIC (3.0 and 7.0 µg/ml) of imipenem and Se-NPs, respectively. The graphical representation in Fig. 3 illustrates distinct patterns of bacterial inhibition that correlate with both concentration and exposure time. At lower concentrations (¼ MIC), the combination maintained approximately 80% inhibition throughout the 24 h with minimal reduction in efficacy over time. The persistence of substantial inhibitory activity (approximately 80%) at the ¼ MIC concentration throughout the 24 h suggests that even at sub-MIC levels, the combination maintains significant antimicrobial activity, which may help prevent the emergence of resistant subpopulations during treatment. The concentration and time-dependent bactericidal action demonstrated in this study provide a basis for designing optimal dosing regimens that could maximize therapeutic efficacy while minimizing the risk of resistance development in the treatment of infections caused by carbapenem-resistant P. aeruginosa. The ½ MIC and ¾ MIC concentrations demonstrated gradual decreases in inhibition percentage, reaching approximately 40% and 30% inhibition, respectively, by the 24-hour timepoint. The most distinct effects were observed with the 1 MIC and 2 MIC combinations, which exhibited rapid decreases in inhibition percentages after 8 h, ultimately reaching complete bacterial eradication at the 20-hour timepoint for 1 MIC and slightly earlier for the 2 MIC concentration. Time-kill kinetics revealed important characteristics of the imipenem-Se-NPs combination against the CRPA isolate. The data demonstrate a concentration-dependent bactericidal effect, with higher concentrations producing more rapid and complete bacterial killing. The observed pattern indicates a biphasic killing curve for the higher concentrations (1 MIC and 2 MIC), characterized by an initial moderate reduction in bacterial population followed by an accelerated killing phase after 8 h of exposure. This biphasic pattern suggests a complex mechanism of action that may involve initial cellular stress followed by cascading antimicrobial effects leading to complete bacterial eradication. The minimum bactericidal concentration (MBC) was identified as the lowest concentration of active compounds capable of eliminating 99.9% of the initial population [60]. Furthermore, the time-kill curve characteristics suggest that maintaining adequate antimicrobial concentrations for at least 20 h would be necessary to achieve complete bacterial eradication at the 1 MIC level.

### Detection of biofilm formation by CRPA isolates

**Fig. 4:**
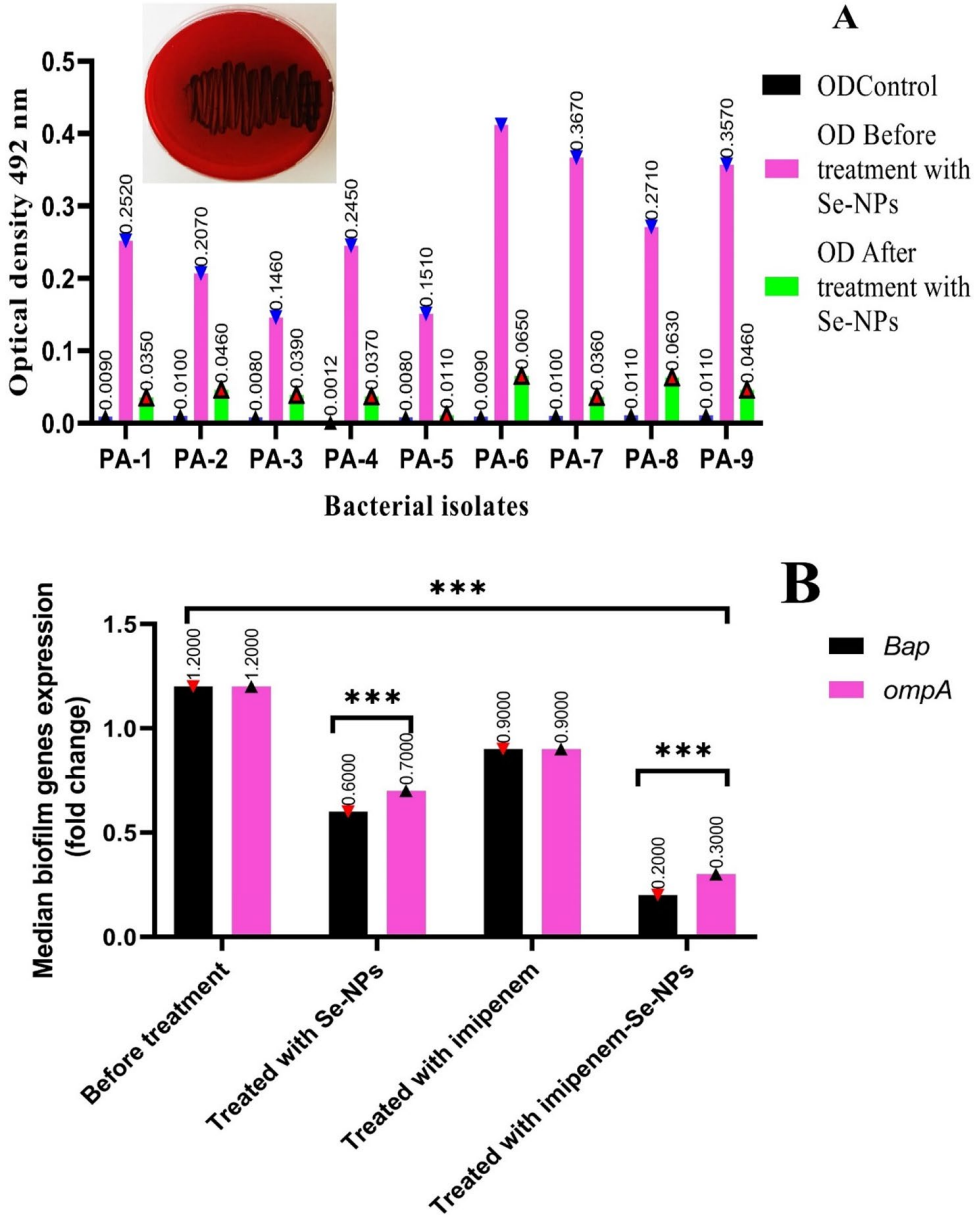
Biofilm inhibition, (**A**) biofilm inhibition study of Se-NPs with crystal violet staining assay (OD = 492 nm), and (**B**) Relative expression *ompA* and of *Bap* genes by quantitative real-time PCR, **p < 0.0028, *** *p* < 0.0001

Initial assessment of biofilm formation capacity among the nine carbapenem-resistant *P. aeruginosa* (CRPA) isolates revealed robust biofilm-producing capabilities across all strains. Using the Congo Red Agar (CRA) method, eight of the nine isolates exhibited rough black colonies, a phenotypic indicator of biofilm production (Fig. 4A). This observation was further substantiated by the microtiter plate assay (MPA), which quantitatively confirmed that all nine isolates were strong biofilm producers, with optical density (OD) values ranging from 0.146 to 0.412. The highest biofilm formation capacity was observed in isolate PA-6 (OD = 0.412), followed by PA-7 (OD = 0.367) and PA-9 (OD = 0.357), while PA-3 exhibited the lowest, though still significant, biofilm formation (OD = 0.146). The consistent demonstration of strong biofilm-forming ability across all tested isolates indicates a critical virulence trait that likely contributes to their carbapenem resistance phenotype and potential persistence in clinical settings.

Molecular characterization through quantitative real-time PCR (qRT-PCR) confirmed the presence of key biofilm-associated genes, *Bap* (biofilm-associated protein) and *OmpA* (outer membrane protein A), in all nine CRPA isolates. The melting curve analysis validated the specificity and efficiency of the primer amplification, confirming the reliability of the gene expression assessment. Following treatment with the sublethal combination of Se-NPs and imipenem at ¾ MIC (2.625 µg/ml and 1.125 µg/ml, respectively), a significant downregulation of both *Bap* and *OmpA* genes was observed in the tested isolate (Fig. 4B). Statistical analysis revealed highly significant reductions in expression levels (*p* < 0.0028 for *OmpA* and *p* < 0.0001 for *Bap*), demonstrating that the combination affects biofilm formation at the transcriptional level. This molecular response correlates directly with the phenotypic changes observed, where the combination treatment completely suppressed biofilm formation as evidenced by the dramatic reduction in optical density values (from 0.146 to 0.412 pre-treatment to 0.009–0.011 post-treatment) and negative results in the CRA test post-treatment.

Perhaps the most remarkable finding from this study is the complete suppression of biofilm formation at sublethal concentrations (¾ MIC) of the Se-NPs-imipenem combination, coupled with the reversal of carbapenem resistance in recovered isolates. Table 8 demonstrates that all nine isolates, which were initially carbapenem-resistant (CR+) and strong biofilm producers, became carbapenem-sensitive (CR-) and lost their biofilm-forming ability after treatment. This phenomenon proposes that the Se-NPs-imipenem combination not only disrupts established biofilms but also fundamentally alters the resistance phenotype of the bacteria. The re-sensitization to carbapenems could be attributed to multiple factors, including the downregulation of *OmpA* (which is associated with membrane permeability and antibiotic resistance) and *Bap* (which contributes to biofilm architecture and stability). The complete suppression of biofilm formation, evidenced by the near-zero OD values (0.009–0.011) in the microplate assay post-treatment, indicates that the combination therapy effectively eliminates this key virulence mechanism. These findings have profound implications for therapeutic strategies against multidrug-resistant infections, signifying that targeting biofilm formation with Se-NPs-imipenem combinations could potentially restore the efficacy of conventional antibiotics against previously resistant strains. The biofilm is a crucial virulence factor governed by the quorum sensing (QS) system in *P. aeruginosa*, which allows for its adherence to host cell surfaces. Various techniques were utilized to detect the biofilm in previous research, including the test tube (TT) method, the Congo red agar method, and the microtiter plate method [40]. A previous study conducted by Nassar et al. [1], showed that 73.4% of *P. aeruginosa* isolates can form biofilms with various degrees. Additionally, research by Da Silva et al. [61], in 2019 found that 86.5% of *P. aeruginosa* isolates can produce biofilm. The remarkable ability of *P. aeruginosa* to form biofilms in diverse environments reduces the efficacy of antibiotic treatments, thereby contributing to the persistence of chronic infections [62]. Furthermore, research by Azizi et al. [63], demonstrated that 23 isolates of *Acinetobacter baumannii*, which exhibited strong biofilm formation, possessed the *ompA* gene in all isolates, while 21 isolates, or 91.14%, contained the *Bap* gene. These findings are consistent with the result reported by Thamayandhi et al. [40], they found that MIC_50_ of selenium nanoparticles completely suppressed biofilm formation by *P. aeruginosa* and downregulated its genes associated with biofilm formation. Biofilms are recognized as critical factors contributing to the emergence of multidrug-resistant bacterial infections. Reports from the National Institutes of Health and the Centers for Disease Control indicate that bacteria that develop biofilms are associated with 65–80% of infections [64]. The presence of bacterial biofilms represents a considerable risk in various fields, particularly in the context of medical devices such as catheters, stents, and implants. Moreover, the formation of biofilms is frequently regarded as a primary factor contributing to the failure of antimicrobial treatments. Given that approximately 65–80% of all infections are believed to be associated with biofilms, this issue poses a significant challenge to effective infection management [64].The selenium nanoparticles demonstrated the capability to inhibit 70% of the mature biofilm biomass of *P. aeruginosa* and *E. coli* at concentrations exceeding 180 µg/ml. The combination of Se-NPs and levofloxacin was applied, and it successfully reduced the biomass of mature biofilms of *S. aureus* and *Enterococcus faecalis* by 70%. Likewise, this combination was able to eradicate 70% of mature biofilm in *P. aeruginosa* and *E. coli* at concentrations of 45:16 µg/mL and 90:32 µg/ml, respectively, when compared to their untreated control groups [38]. In addition, Fareid and his team [65], utilized nanoparticles to inhibit the expression of genes associated with biofilm formation, achieving a decrease in the levels of the *icaA* and *icaD* genes by 1.9 to 2.2-fold and 2.4 to 2.8-fold, respectively.

**Table 8:** Detection of biofilm formation and its genes pre- and post-treatment

Isolated bacteria	Biofilm formation								
	Before treatment						After treatment with IMP-Se-NPs		
	CRA Test	MPA OD	Class	Biofilm genes		CR	CRA	MPA OD	CR
				*ompA*	*Bap*				
PA-1	**+**	0.252	Strong	**+**	**+**	**+**	**-**	0.010	**-**
PA-2	**+**	0.207	Strong	**+**	**+**	**+**	**-**	0 0.010	**-**
PA-3	**+**	0.146	Strong	**+**	**+**	**+**	**-**	0.009	**-**
PA-4	**+**	0.245	Strong	**+**	**+**	**+**	**-**	0.009	**-**
PA-5	**+**	0.151	Strong	**+**	**+**	**+**	**-**	0.009	**-**
PA-6	**+**	0.412	Strong	**+**	**+**	**+**	**-**	0.009	**-**
PA-7	**+**	0.367	Strong	**+**	**+**	**+**	**-**	0.010	**-**
PA-8	**+**	0.271	Strong	**+**	**+**	**+**	**-**	0.011	**-**
PA-9	**+**	0.357	Strong	**+**	**+**	**+**	**-**	0.011	**-**

CRA = Congo red agar, MPA = Microplate technique, OD = Optical Density, CR = carbapenem resistance

### In-vitro cytotoxicity of biosynthesized Se-NPs, imipenem-Se-NPs against normal and cancer cells

**Fig. 5:**
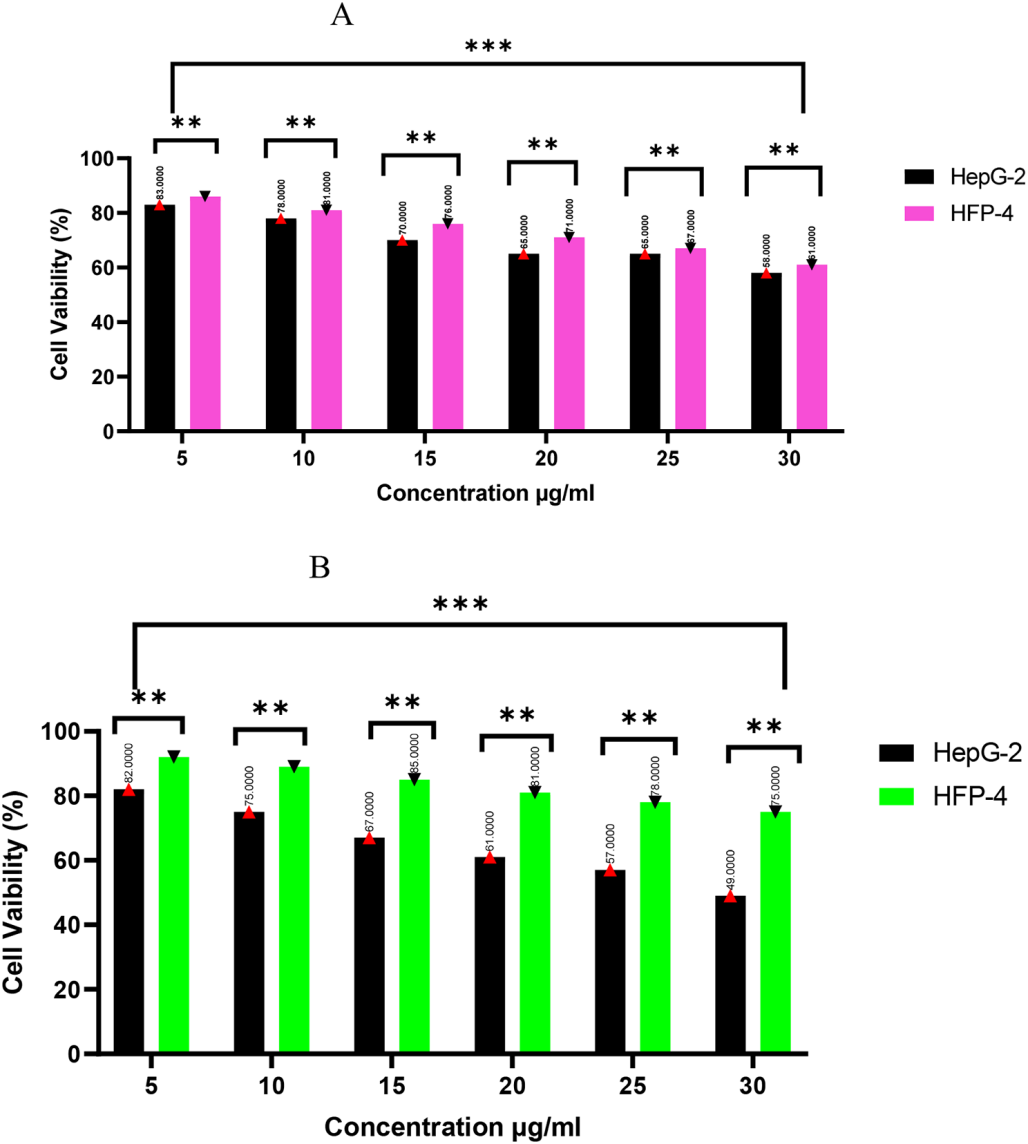
In vitro cytotoxicity study (**A**) Se-NPs, (**B**) imipenem -Se-NPs (MIC=1.5+3.5 μg/μg/ml, respectively); 5= 0.5 MIC, 10=1, 15=1.5, 20=2, 25= 2.5 and 30= 3MIC against HFP-4 and HepG-2 cells, **p < 0.0028, *** *p* < 0.0001

The in vitro cytotoxicity assessment of Se-NPs demonstrated a concentration-dependent effect on both HFP-4 (normal human melanocytes) and HepG-2 (hepatocellular carcinoma) cell lines across the tested concentration range (5–30 µg/ml). As shown in Fig. 5A, cell viability gradually decreased with increasing Se-NPs concentration in both cell lines. At the lowest concentration (5 µg/ml), both cell lines maintained high viability (86.7 ± 1.21% for HFP-4 and 83.3 ± 1.12% for HepG-2), indicating minimal cytotoxic effects at this dosage. As concentrations increased to 30 µg/ml, viability declined to 61.2 ± 1.14% and 58.4 ± 2.12% for HFP-4 and HepG-2 cells, respectively. The statistical analysis revealed significant differences between concentration groups (*p* < 0.0001), confirming the dose-dependent nature of Se-NPs cytotoxicity. Remarkably, while both cell lines showed decreasing viability with increasing concentration, the cytotoxic effect was marginally more obvious in HepG-2 cancer cells compared to normal HFP-4 cells at most concentrations, though this differential effect was modest. The combination of imipenem and Se-NPs (at a fixed ratio based on their MIC value of 1.5 + 3.5 µg/ml) exhibited a markedly different cytotoxicity profile compared to Se-NPs alone, as illustrated in Fig. 5B. The combination therapy demonstrated enhanced selectivity toward cancer cells, with HepG-2 cells showing significantly lower viability compared to normal HFP-4 cells across all tested concentrations. At the highest concentration tested (3 MIC, equivalent to 4.5 + 10.5 µg/ml of imipenem + Se-NPs), HepG-2 cell viability decreased to 49.6 ± 3.14%, while HFP-4 cells maintained considerably higher viability at 75.6 ± 1.14%. This differential effect was consistent across all concentration points, with normal cells consistently showing 20–30% higher viability than cancer cells. The statistical analysis confirmed significant differences between both concentration groups and cell types (*p* < 0.0005), emphasizing both the dose-dependent effect and the selective targeting of cancer cells by the combination treatment.

The IC50 values provide valuable insights into the relative safety of these treatments. Se-NPs exhibited IC50 values of 52.4 µg/ml for HFP-4 (normal cells) and 32.2 µg/ml for HepG-2 (cancer cells), indicating a moderate degree of selective toxicity toward cancer cells. This selectivity index (ratio of IC50 in normal cells to cancer cells) of approximately 1.63 suggests a favorable therapeutic window. When considering the antimicrobial applications of Se-NPs, these findings are particularly significant as they demonstrate that the concentrations effective against carbapenem-resistant *P. aeruginosa* (MIC of 14 µg/ml for Se-NPs alone and 3.5 µg/ml in combination with imipenem) are well below the cytotoxic thresholds for normal human cells. The imipenem-Se-NPs combination further improves this therapeutic profile by maintaining even higher viability in normal cells while effectively targeting cancer cells. This dual-action profile antimicrobial efficacy coupled with potential anticancer activity and relative safety toward normal cells emphasizes the promising therapeutic potential of these nanoparticle-based treatments.

The observed cytotoxicity profiles can be explained by several potential mechanisms. Selenium nanoparticles are known to induce oxidative stress through the generation of reactive oxygen species (ROS), which can damage cellular components including proteins, lipids, and DNA. Cancer cells often exhibit altered redox status and impaired antioxidant defense mechanisms compared to normal cells, potentially explaining the preferential toxicity toward HepG-2 cells. The enhanced selective effect of the imipenem-Se-NPs combination suggests synergistic interactions that may amplify this differential response. In a previous study by Long et al. [66], the cytotoxic effects of selenium nanoparticles (Se-NPs) were assessed in vitro using the HepG-2 cell line at four specific concentrations: 0, 5, 10, 15, and 20 µg/ml. The findings demonstrated that the viability of HepG-2 cells varied from 83.9 ± 1.58–66.4% ± 6.94%. Significant differences in cell viability were observed among the tested concentrations (*p* < 0.05). Furthermore, Recent findings suggest that the green synthesis of selenium nanoparticles utilizing garlic (*Allium sativum*) pulp extract exhibited minimal cytotoxicity at various concentrations (15, 30, 60, and 90 µg mL − 1) when assessed on Vero cells [67]. Similarly, Se-NPs synthesized from lemon (*Citrus limon*) leaf extract demonstrated a very low toxic effect on human lymphocytes at a concentration of 0.1 µg µl^− 1^ [68]. Additionally, Se-NPs based on *Spirulina polysaccharides* showed negligible cytotoxicity towards human normal kidney HK-2 cells at a concentration of 450 µM over 72 h, while a low cytotoxic effect was noted against Caco-2 mammalian cells at concentrations ranging from 10 to 40 µg/ml [69]. In this context, Se-NPs were synthesized using the cell-free filtrate of *Idiomarina* sp. PR58-8 demonstrated no cytotoxicity towards the human epidermal keratinocyte cell line, HaCaT, at concentrations ranging from 5 to 100 µg/ml [70].

### Haemolysis activity

**Fig. 6:**
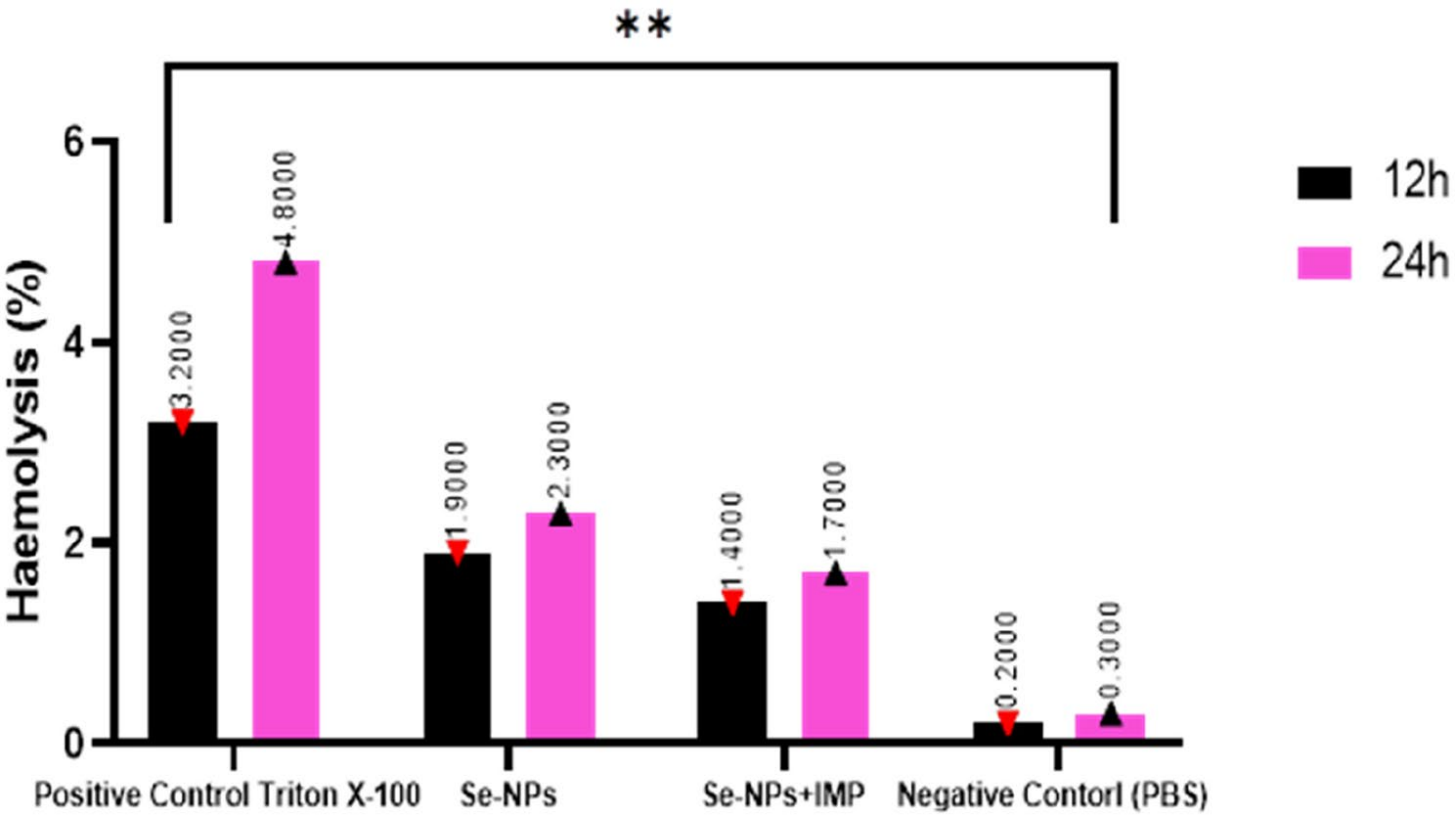
Hemolytic activity of Se-NPs and their combination with imipenem. **(*p* < 0.0161)

The haemolytic profile of Se-NPs and its combination with imipenem was evaluated at MIC levels over 12 and 24-hour exposure periods, providing critical insights into their hemocompatibility for potential clinical applications. As illustrated in Fig. 6, all tested treatments exhibited time-dependent increases in haemolytic activity. Triton X-100, serving as the positive control, demonstrated the highest haemolytic potential with values of 3.2% and 4.7% at 12 and 24 h, respectively, establishing the upper border for the haemolysis assay. In contrast, Se-NPs administered alone showed moderate haemolytic activity, with haemolysis percentages of 1.9% at 12 h and 2.3% at 24 h. Notably, when Se-NPs were combined with imipenem, the haemolytic activity was reduced to 1.4% and 1.7% at 12 and 24 h, representing approximately 26% and 26% reductions in haemolysis compared to Se-NPs alone at the respective time points. The negative control (PBS) exhibited minimal haemolysis (0.2–0.3%), confirming the assay’s specificity and establishing the baseline for the experiment. Statistical analysis revealed significant differences (*p* < 0.0161) between the treatment groups, particularly between the positive control and the other test conditions. The observed reduction in haemolytic activity when selenium nanoparticles are combined with imipenem suggests important mechanistic interactions that enhance the hemocompatibility of the combination therapy. Several factors may contribute to this improved safety profile. First, the surface properties of Se-NPs may be modified in the presence of imipenem, potentially reducing direct interactions with erythrocyte membranes. Second, imipenem may compete with Se-NPs for binding sites on red blood cell surfaces, thereby limiting the nanoparticles’ capacity to disrupt membrane integrity. Third, the combination might reduce the generation of reactive oxygen species (ROS) typically associated with nanoparticle-induced haemolysis. The haemolysis percentages for all tested formulations remained below 5%, which is generally considered acceptable for intravenous formulations according to standard hemocompatibility guidelines. Blood functions as the primary transport for NPs introduced through intravenous methods while also acting as the pathway for NPs administered through other routes to reach their target tissues or organs. The size of NPs allows for extensive distribution within the body, enabling them to traverse biological barriers and enter systemic circulation, thereby facilitating their effective infiltration into cells [71]. Furthermore, the small size of NPs enhances their biological activity compared to larger particles, which can disrupt the normal biochemical environment within cells, As a result, the interactions between NPs and blood components are not only necessary but may also present considerable risks, thereby rendering hemocompatibility an essential factor in the design and development of NPs intended for therapeutic applications [72]. The application of Se-NPs in internal medicine depends on their ability to exhibit minimal or no hemolytic activity. The hemolytic activity of the majority of nanoparticles is influenced by factors such as concentration, structure, size, and shape [71]. Yazhiniprabha et al. [73], investigated the hemolytic activity of Se-NPs using goat erythrocytes and observed it with an inverted research microscope, their finding indicated an absence of hemolytic activity and suggested that the various concentrations (25, 50, 75, and 100 µg/ml) of Se-NPs did not result in any changes to the erythrocytes. Nonetheless, a slight disruption of the erythrocyte membrane was observed at the highest concentration of 100 µg/ml. Additionally, Se-NPs exhibited minimal haemolytic properties at the maximum concentration of 128 ppm against horse blood erythrocytes. Similarly, crocin-conjugated PEG-Se-NPs did not cause any changes in treated human erythrocytes across systematically varied concentrations [74].

## Conclusion

Carbapenem-resistant *P. aeruginosa* infections, which arise from biofilm formation and/or acquired resistance, necessitate the creation of unconventional and innovative drug strategies. Selenium nanoparticles present a variety of benefits for human health. Their environmentally friendly synthesis is noted for being safer, more straightforward, quicker, and more economical than traditional physicochemical approaches. After confirming the biosynthesis of these nanoparticles using *Streptomyces* sp., susceptibility testing, specifically the MICs, reveals that these nanoparticles can effectively inhibit CRPA growth at lower concentrations than standard antibiotic treatments and impede its biofilm formation. Combining Se-NPs with imipenem showed a synergistic effect with a significant reduction in the MIC of Se-NPs and imipenem as well as downregulation of the *Bap* and *ompA* genes. As a result, the use of these nanoparticles may help reduce the emergence of antibacterial resistance and provide a promising alternative for addressing this challenge in the future. Additionally, this scenario paves the way for investigating the potential of nanoparticles against other microorganisms, such as other bacteria species, fungi, parasites, and viruses, as well as for the diagnosis and treatment of related diseases.

### Limitations and prospects of the study

The authors recognize the limitations inherent in their study but express optimism regarding the efficacy of selenium nanoparticles, whether used independently or in conjunction with imipenem formulations, in tackling the challenges presented by CRPA. However, several significant hurdles must be addressed. The restricted number of bacterial targets complicates the ongoing quest for entirely new compounds aimed at innovative targets. Furthermore, the risk of resistance development poses a substantial threat to these novel agents. Therefore, the combination of nanocarriers with established antibiotics may not only mitigate the rapid emergence of resistance and virulent bacterial factors but also enhance pharmacokinetics and targeting precision. A holistic approach that encompasses the creation of new compounds alongside the improvement of existing therapies through nanocarrier technologies is essential for ameliorating the current precarious situation. Nevertheless, achieving successful outcomes will necessitate collaborative efforts among policymakers, academic institutions, and the pharmaceutical sector.

## Data Availability

All data generated or analyzed during this study are included in this published article.
